# Adolescent rats engage the orbitofrontal-striatal pathway differently than adults during impulsive actions

**DOI:** 10.1038/s41598-024-58648-w

**Published:** 2024-04-13

**Authors:** Aqilah M. McCane, Lo Kronheim, Alejandro Torrado Pacheco, Bita Moghaddam

**Affiliations:** Oregon Health and Science University, Portland, OR USA

**Keywords:** Developmental biology, Neuroscience, Diseases

## Abstract

Adolescence is characterized by increased impulsive and risk-taking behaviors. To better understand the neural networks that subserves impulsivity in adolescents, we used a reward-guided behavioral model that quantifies age differences in impulsive actions in adult and adolescent rats of both sexes. Using chemogenetics, we identified orbitofrontal cortex (OFC) projections to the dorsomedial striatum (DMS) as a critical pathway for age-related execution of impulsive actions. Simultaneous recording of single units and local field potentials in the OFC and DMS during task performance revealed an overall muted response in adolescents during impulsive actions as well as age-specific differences in theta power and OFC–DMS functional connectivity. Collectively, these data reveal that the OFC–DMS pathway is critical for age-differences in reward-guided impulsive actions and provide a network mechanism to enhance our understanding of how adolescent and adult brains coordinate behavioral inhibition.

## Introduction

Adolescence is a developmental period associated with risky behaviors and increased sensation seeking. While these phenotypes may be adaptive and help adolescents acquire new skills and facilitate independence, they may lead to impulsivity and poor decision making. Our knowledge of neural networks that subserve impulsivity in adolescent models remains limited^1,2^. Impulsive actions may directly affect the ability to withhold a response that was previously rewarding, a behavior known as response inhibition. Response inhibition deficits are greater in adolescents than adults^3,4^. Understanding the neural mechanisms of response inhibition in adolescents is critical for identifying those most at risk for reckless and impulsive decision making^5^.

The developmental period of adolescence to early adulthood is associated with considerable neuronal maturation in frontal cortical regions such as the orbitofrontal cortex (OFC) and in striatal regions^6,7^. In adults, these regions have been implicated in response inhibition and impulsive actions. For example, response inhibition is associated with activation of the OFC in healthy adults^8^, and a dysfunctional OFC activity is observed in patients with disorders characterized by deficits in response inhibition such as obsessive compulsive disorder, Tourette’s syndrome and attention deficit hyperactivity disorder^9–12^. In preclinical rodent models, OFC lesions impair response inhibition^13,14^, whereas optogenetic stimulation of the OFC reduces spontaneous compulsive behaviors^15^. While OFC projects to most striatal subregions, its projections to the dorsomedial striatum (DMS)^16^ are hypothesized to mediate response inhibition^17^. This notion is supported by optogenetic stimulation of this pathway reversing deficits in response inhibition in a rodent model of compulsive behavior^15^ and DMS lesions impairing behavioral inhibition similar to prefrontal cortex damage^18^. Finally, in adolescents, OFC and DMS neurons encode the same rewarded actions differently than adults^19,20^.

Here we asked if OFC–DMS projections process impulsive actions differently in adolescents and adults. Sparsity of preclinical data related to neural processing of behavioral inhibition in adolescents is primarily due to limitations caused by the brief period of adolescence in rodents where behavioral and in vivo measures have to be completed in less than two weeks. We overcame these limitations by developing the cued response inhibition task (CRIT), an operant task which measures response inhibition in both adolescents and adults^21^ using the same training duration. Here we combined this behavioral model with chemogenetics or electrophysiological recording of units and local field potentials (LFPs) and observe age-dependent roles for OFC–DMS projections to support impulsive actions.

## Results

### OFC to DMS projection is critical for expression of response inhibition in an age-specific manner

**Figure 1:**
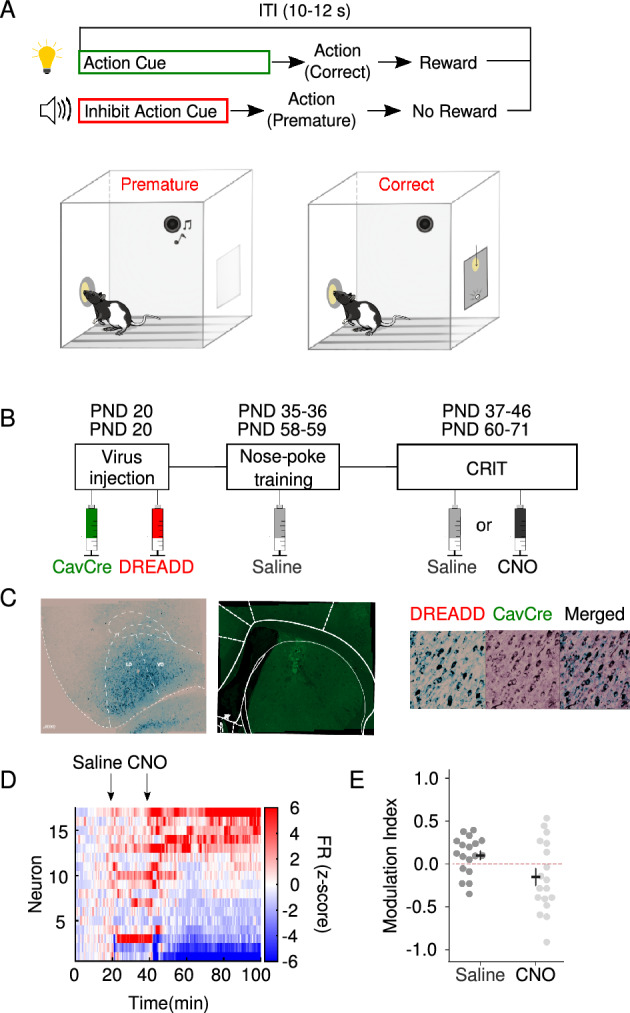
Experimental methods. (**A**) Experimental design of the cued response inhibition task (CRIT). Sessions begin with simultaneous presentation of an inhibit action cue (variable time 5–30 s), and an action cue which remained lit for 10 s following cessation of the inhibit action cue. Created with Biorender.com. (**B**) Schematic of chemogenetic methods. DREADDs receptors were expressed in DMS projecting OFC neurons by injecting CAV/2 Cre (green) bilaterally in the DMS and inhibitory DREADDs receptors (red) in the OFC. (**C**) Robust expression of both viruses in their respective brain regions is observed. (**D**) Administration of CNO (10 mg/kg) suppresses firing rate (Z-scored) in a spontaneously active population of OFC neurons (not limited to DREADDs expressing units) under isoflurane anesthesia. (**E**) Compared to saline control (light grey), CNO injection (dark grey) produced a significant and sustained modulation of OFC firing rate.

To assess the role of OFC–DMS projections in response inhibition, we quantified premature responses during CRIT training (Fig. 1A) while inhibiting OFC cells that project to DMS using chemogenetics. We infused a Cre virus (Cav2Cre) into the DMS and an inhibitory DREADD (designer receptors exclusively activated by designer drugs; AAV5-hSyn-DIO-hM4D(Gi)-mCherry) into the OFC (Fig. 1B,C). To validate the effectiveness of this approach to influence OFC neuronal activity, we performed in vivo electrophysiology recordings and observed significant modulation of OFC firing rate following clozapine-N-oxide (CNO) administration (t = 2.31, *p* = 0.03: Fig. 1D, E) compared to saline. Moreover, to control for nonspecific effects of virus or CNO injections, we performed control experiments using a control virus (AAV-hSyn-DIO-mCherry) injected into the OFC and Cav2Cre into the DMS as well as treated surgery naïve animals with CNO (Supplemental Figure 1).

**Figure 2:**
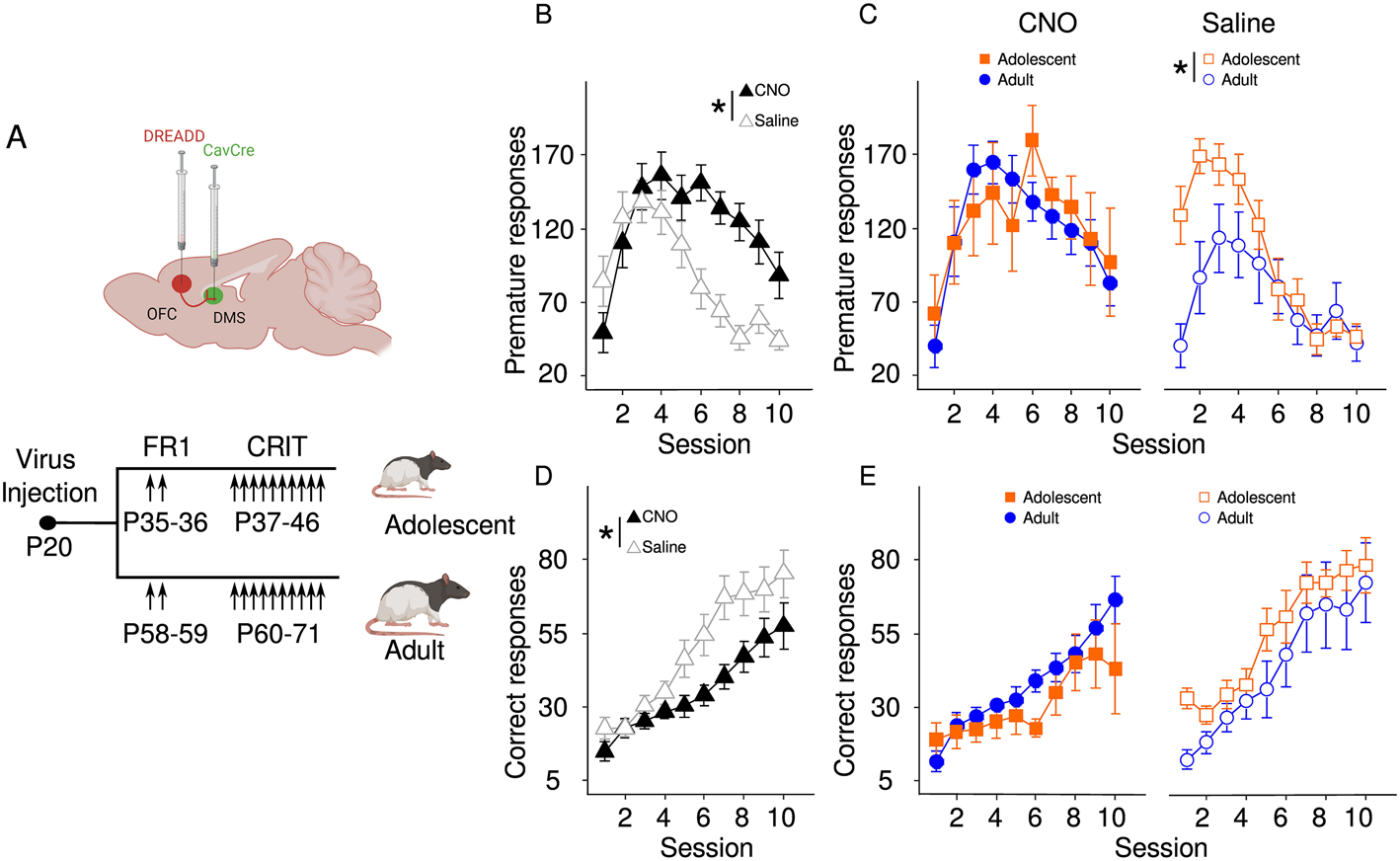
Chemogenetic manipulation of corticostriatal circuits. (**A**) Timeline of CRIT showing animals’ age at each stage for adolescents (top) and adults (bottom). Arrows represent days animals were injected with either saline or CNO. Created with Biorender.com. (**B**) CNO (10 mg/kg) treatment resulted in increased premature actions in both age groups during later sessions. (**C**) Number of premature actions is similar between adults and adolescents treated with CNO, but adolescent premature actions differ from adults across sessions following saline treatment. (**D**) CNO treatment resulted in reduced correct actions in both age groups. (**E**) Both adolescents and adults increase the number of correct actions across session. Data are presented as mean + SEM. *p < 0.05 main effect.

We next measured the effect of inhibition of DMS projecting OFC cells in adults and adolescents during CRIT by injecting animals with CNO (10 mg/kg) or saline for 10 consecutive days 30 min before behavioral testing. All animals were injected with saline during fixed ratio 1 (FR1) training to become habituated to injections (Fig. 2A). Differences were observed in premature actions (Fig. 2B). As CRIT training progressed, premature actions initially increased [main effect of session: F(9,221) = 11.29, *p* = 1.70 × 10^–14^] as correct actions also increased [main effect of session: F(9,221) = 46.63, *p* < 2 × 10^–16^]. CNO injection, as compared to saline, significantly increased premature actions [main effect of group: F(1,24) = 10.52, *p* = 0.003; Fig. 2B] suggesting that OFC projections to DMS play a key role in behavioral inhibition. Critically, an age-related difference emerged when we compared the effect of CNO and saline in adolescents vs adults. Whereas the number of premature actions in the saline control group were influenced by both age and session [age by session interaction: F(9,108) = 3.36, *p* = 0.001], with adolescents performing more premature actions than adults, the CNO adult group became more adolescent-like in that inhibition of DMS projecting OFC cells resulted in a statistically similar amount of premature actions in adolescents and adults [main effect of age: F(1,7) = 0.22, *p* = 0.66; Fig. 2C]. The number of correct trials was similarly reduced in both age groups after CNO administration [main effect of group: F(1,24) = 5.40, *p* = 0.03; Fig. 2D—main effect of session: F(9,108) = 31.004, *p* < 2 × 10^–16^; Fig. 2E]. Collectively these data indicate that while OFC cells that project to the DMS are critical for CRIT performance in both groups, silencing this pathway ablates age differences in response inhibition.

### Adolescent OFC and DMS exhibit different electrophysiological correlates to response inhibition

**Figure 3:**
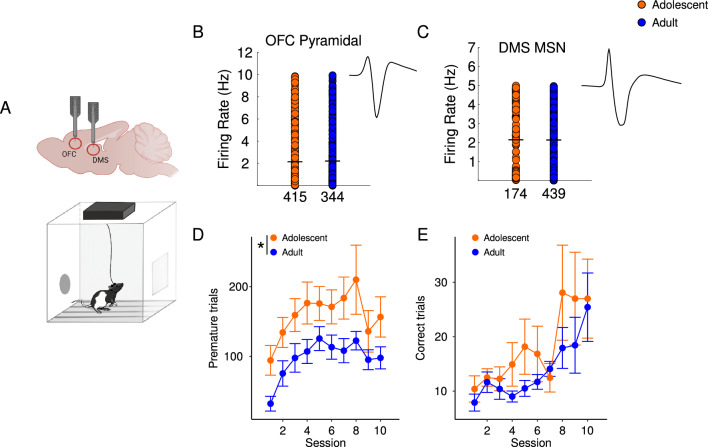
Simultaneous recording from OFC and DMS in adults and adolescents as they perform CRIT (**A**) Schematic of the operant chamber where simultaneous recordings in the OFC and DMS were performed. Created with Biorender.com. (**B**) Baseline firing rate in adolescent (orange) and adult (blue) putative pyramidal cells (**C**), and medium spiny neurons (MSNs;2). Numbers reflect total number of cells recorded across all sessions. There were no age differences baseline firing rate. (**D**, **E**) Behavioral data for both age groups performing CRIT. (**D**) Adolescents make more premature responses during CRIT. (**E**) Adolescents and adults made similar number of correct responses. Data are presented as mean + SEM. *p < 0.05, main effect of age.

Given the critical role of OFC and DMS in response inhibition and observed age-related behavioral differences, we investigated the neural correlates of relevant behavioral events in CRIT while recording units in adults and adolescents (Fig. 3A). We classified cells as putative pyramidal cells or medium spiny neurons based on their firing rates and spike widths. Baseline firing rate in the OFC and DMS was statistically similar between adolescents and adults (independent samples t-test: *p* values > 0.05, Fig. 3B,C). Consistent with untethered animals (Fig. 2), adolescents made more premature responses than adults during CRIT [main effect of age: F(1,21) = 6.27, *p* = 0.02; Fig. 3D] while the number of correct trials completed was comparable between age groups [main effect of age: F(1,16) = 0.38, *p* = 0.55; Fig. 3E]. Adolescents also executed more total actions compared to adults [main effect of age: F(1,20) = 5.43, *p* = 0.03; supplemental Figure 2].

**Figure 4:**
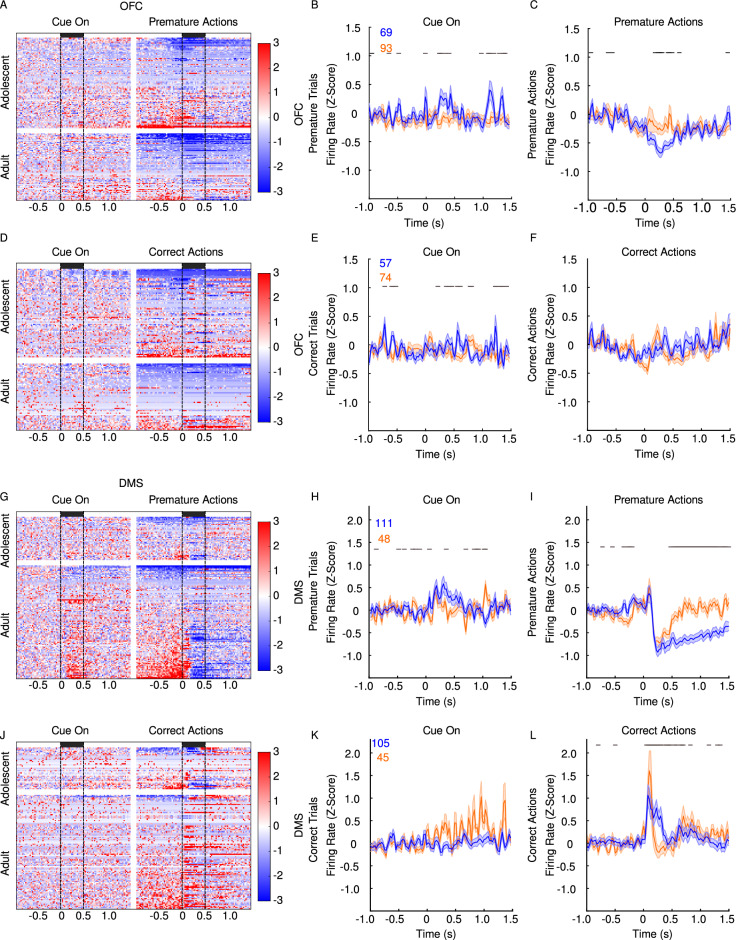
Single unit electrophysiology recordings in OFC and DMS during cue presentation, premature action and correct actions in adults and adolescents. (**A**–**F**) OFC heatmaps for unit firing and corresponding mean firing rate (**G**–**L**). DMS heatmaps for unit firing and corresponding mean firing rate. The bars on the heatmaps in the left column depict the 500 ms change in firing rate following the task event (cue on, or action). Age differences in line graphs on the right (Z-score responses) are depicted as adults in blue and adolescents in orange. The panel events (tone on, premature or correct action) occurred at time 0 s. Adults (blue) but not adolescents (orange) show a suppression of OFC cell firing rate after premature responding (**A**). In the DMS, MSNs show age differences in response to premature responding (**B**). OFC cell firing rate was not different between adults and adolescents following correct responses (**C**). Adolescent DMS MSNs show a larger response to reward compared to adults (**D**). Numbers at the top of y-axis reflect cell counts for each group. Data are presented as mean + SEM. Black significant bars reflect permutation testing between age groups, *p* < 0.05.

We next investigated the physiological correlates of behavior in both age groups by isolating the firing activity of single units in the OFC and DMS. Data presented are aggregated from the last three days of recording (PND 44–46 and 69–71). While some age-related small differences were observed during tone exposure in either region, the more robust phasic response differences were observed during action (premature or correct) execution. In response to premature actions, while different populations of OFC putative pyramidal cells in both age groups were inhibited or excited (Fig. 4A), the global firing rate was significantly decreased only in adults (Fig. 4C). In contrast, no differences between adolescent and adult OFC firing rates were observed after correct responses (Fig. 4F). In the DMS, while both adolescents and adults show a suppression of MSN firing rates after premature responding, this effect was more persistent in adults (Fig. 4G–I). Compared to adults, adolescents exhibited a different response to correct actions and the resulting reward in DMS MSNs (Fig. 4L). While neurons in both groups displayed a phasic response, the temporal profile of this response was different in each age group.

### Local field potential recordings reveal age-specific alterations in the theta oscillations

To further understand the neural correlates of CRIT, we also analyzed LFP activity in the OFC and DMS of the same animals depicted in Figs. 3 and 4. Power spectral densities were parametrized using the FOOOF toolkit^22^. We focused our analysis on the theta frequency band (5–11 Hz) because this frequency is associated with cognitive control^23,24^, response inhibition^25–27^, and reward processing^28,29^. LFP analyses included power, aperiodic exponent and phase synchrony.

**Figure 5:**
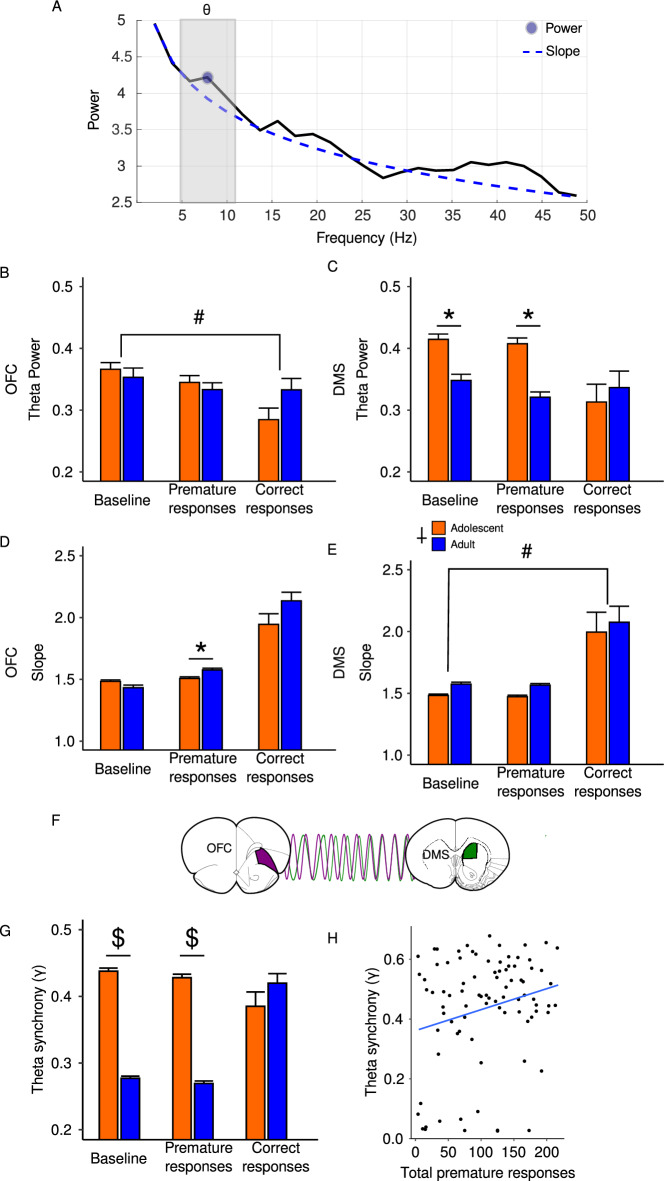
Simultaneous local field potential recordings in OFC and DMS in adults and adolescents during CRIT performance. (**A**) Schematic of LFP methods for determining age differences in LFP measures. Theta power, aperiodic exponent (power spectral density slope) and theta phase synchrony during task performance in the OFC and DMS were isolated from the power spectrum density in adults and adolescents (please see methods for detail). (**B**, **C**) Compared to baseline, OFC theta power is reduced following correct actions. Adolescents exhibit greater DMS theta power than adults at baseline and following premature actions. (**D**, **E**) The aperiodic exponent in OFC and DMS. The exponent was larger in adults than adolescents in the OFC during premature actions. In the DMS, the aperiodic exponent was increased following correct actions and was larger in adults compared to adolescents for all events. (**F**, **H**) Schematic and results of phase synchrony between the OFC and DMS. Phase synchrony between the OFC and DMS was stronger in adolescents compared to adults at baseline and during premature actions (**G**). Synchrony during premature actions in both age groups was also correlated with the total number of premature responses (**H**). Data are presented as mean + SEM. #*p* < 0.05, Tukey Post hoc, *p < 0.05 Bonferroni post hoc, *p* < 0.05, main effect of age, $*p* < 0.05, Welch’s two sample t-test.

Mean theta power was extracted from putative oscillatory activity (Fig. 5A). OFC theta power was significantly influenced by event [main effect of event: F(2,560) = 5.11, *p* = 0.006], where power during correct actions was reduced, relative to baseline [Tukey Post hoc, *p* = 0.007; Fig. 5B]. There was no effect of age on OFC theta power across events [main effect of age: F(1,560) = 0.27, *p* = 0.61]. In the DMS, theta power was significantly influenced by both event and age [event by age interaction: F(2,767) = 3.15, *p* = 0.04; Fig. 5C]. Compared to adults, adolescents exhibited stronger DMS theta power at baseline (p = 2.12 × 10^–6^, Bonferroni post hoc) and following premature actions (p = 1.04 × 10^–10^, Bonferroni post hoc; Fig. 5C).

The aperiodic exponent or power spectrum density slope was extracted and used as an index of excitation-inhibition balance^30^. Steeper slopes or larger exponents are hypothesized to reflect greater inhibition, while flatter slopes or smaller exponents occur when excitation is greater than inhibition^30^. There was a significant event by age interaction for aperiodic exponents in the OFC [F(2,506) = 9.01, *p* = 0.0001], followed with Bonferroni corrected post hoc tests. Adolescents had smaller exponents in the OFC during premature actions (*p* = 0.001, Bonferroni post hoc; Fig. 5D). In the DMS, adolescent aperiodic exponents were lower than adults across all events [main effect of age: F(1,767) = 47.81, *p* = 9.89 × 10^–12^; Fig. 5E]. Aperiodic exponents were also significantly influenced by event [main effect of event: F(2,767) = 104.64, *p* < 2 × 10^–16^], where both ages exhibited larger aperiodic exponents following correct actions (*p* < 0.001, Tukey post hoc).

Finally, we assessed functional connectivity between the OFC and DMS in the theta frequency band by computing the phase locking index γ as reported previously^31^ (Fig. 5F). Adolescents exhibited stronger OFC–DMS synchrony during baseline [t(2591.6) = 28.50, *p* < 2.2 × 10^–16^] and after premature responding [t(1979.8) = 24.62, *p* < 2.2 × 10^–16^; Fig. 5G]. Higher theta synchrony was positively correlated with total premature actions r(85) = 0.23, *p* = 0.03 (Fig. 5H).

## Discussion

A wealth of adult-focused literature implicates cortico-striatal circuits in mediating response inhibition^17,32,33^. The nature of the contribution of these cortico-striatal networks and circuits to adolescent impulsivity and response inhibition is poorly understood. We have previously observed age-related differences in reward processing in the OFC and DMS^19,20^. We therefore hypothesized that the immaturity of adolescent cortical-striatal circuits may underlie these age differences in impulsivity. To test this hypothesis, we used a response inhibition task, where adolescents learn similarly to adults to withhold an action to receive a reward^21^, but make more premature actions than adults. We find that while chemogenetic inhibition of OFC–DMS pathway increases impulsive actions in both ages, it has a more robust influence in adults making them more adolescent-like. The analyses of unit recording form OFC and DMS further supported our hypothesis by demonstrating a different pattern of neural engagement and coordination in these regions in adults and adolescents.

### OFC–DMS projection is critical for expression of impulsive actions

The OFC is strongly implicated in response inhibition. Metabolic activity in the OFC is related to compulsivity and impulsivity in Tourette’s syndrome^9^. Increased activation of OFC is observed when healthy adults are engaged in response inhibition^8^ whereas reduced OFC activation is observed when response inhibition is impaired in individuals with OCD^10,12^ or ADHD^11^. In rodent models, OFC lesions are associated with increased impulsive choices^34^, impaired response inhibition^13,14^ and impaired reversal learning^35,36^. Additionally, optogenetic stimulation of the OFC reduces spontaneous compulsive behaviors^15^. Collectively this literature demonstrates that aberrant activity in the OFC across a spectrum of disorders, and in both clinical and preclinical models, is associated with compulsive and impulsive responding.

The OFC projects to the DMS^16^, and this pathway is hypothesized to play a role in compulsive behaviors^37^. Similar to the OFC, the DMS plays a role in response inhibition^17,38–40^ and goal-directed actions^41–43^. The OFC influences actions via direct modulation of the DMS^44^. Lesions of DMS impaired behavioral inhibition similar to prefrontal cortex damage^18^, suggesting that activity in both of these regions is necessary for expression of response inhibition**.** Consistent with this notion, optogenetic stimulation of the OFC-striatum pathway reverses deficits in response inhibition in a rodent model of compulsive behavior^15^.

Our findings here complement the above adult-focused literature by demonstrating that the OFC projections to DMS are necessary for appropriate expression of response inhibition. Our results further demonstrate that the OFC–DMS projection has a more robust influence in controlling response inhibition in adults. Specifically, the observation that chemogenetic inhibition of OFC–DMS makes adults’ response inhibition more adolescent-like provides strong evidence that maturation of this pathway is critical for better response inhibition in adults.

### Adolescents OFC and DMS neurons are engaged differently during CRIT

OFC population activity significantly decreased after premature actions in adults but not adolescents. Previous work in adult models has shown that OFC activity before an action execution is modulated by previous actions and predicts subsequent response duration, suggesting that the OFC may use previous action-related information to influence current actions^45^. Consistent with this function of the OFC, we observed that OFC activity was significantly altered after premature actions in adults but not in adolescents. These age differences further suggest that actions informed by action-history develop with maturity. Adolescents may, therefore, have reduced capacity to utilize prior events to inform current events, likely due to functionally immature cortical networks. Alternatively, age differences in behavioral performance and corresponding cortical-striatal dynamics may reflect impaired acquisition of the CRIT. The OFC is hypothesized to guide decision making via state representation or the formation of a cognitive map^46–48^. As such, inactivation of the OFC disrupts learned behavioral performance^48,49^, possibly via disruption of cognitive map formation. Therefore, inhibition of OFC–DMS cells in an immature OFC network may result in a different pattern of cognitive mapping during task performance in adolescence.

DMS neurons are influenced by the OFC and inactivation of the OFC impairs DMS state representation^44^ and decision-making^50^. In our single unit data, strong age differences were observed in the DMS after premature actions where both adult and adolescent firing rates decreased. The temporal profile of this phasic response was different in that adult neurons remained inhibited longer whereas adolescent neurons return to baseline quickly, possibly reflecting weaker cortical inhibition in adolescents. Consistent with previous findings^20^, we observe age differences in the DMS after correct actions with adolescents showing a stronger phasic response to reward compared to adults. Post-action striatal responses may reflect encoding of action outcome values^45,51^. These values may be updated from trial to trial, and in this way influence future responding. In adolescents, impaired encoding of outcomes may result in a failure to utilize feedback to guide future behaviors resulting in persistent premature actions. The DMS is hypothesized to encode reward value^51,52^, which can also influence future actions. Moreover, DMS-projecting OFC neurons play a role in encoding reward value^50^. Therefore, a larger DMS response in adolescents to reward may suggest that they assign greater value to reward than adults.

### Adolescent OFC and DMS network dynamics are different during CRIT

Coordinated changes in neuronal activity give rise to changes in net current flux and LFP oscillations^53^. These oscillations may provide information about event-driven or state-level global activity in the brain. Importantly, LFPs cannot disambiguate the contributions of individual neurons but their activity may be an indirect measure of network level dynamics^54^. We recorded LFPs in adults and adolescents during CRIT and utilized the fitting oscillations and one over f (FOOOF) algorithm^22^.This algorithm extracts the aperiodic component which is hypothesized to reflect the balance between excitation and inhibition. Specifically, when excitation is greater than inhibition, the exponent is lower^30,55^. The OFC aperiodic exponent in adolescents was lower than adults during premature actions, indicating less cortical inhibition. Reduced inhibition may be associated with impulsivity in clinical populations. For example, adolescents with ADHD, a disorder characterized by increased impulsivity and reduced response inhibition, have smaller exponents than control adolescents^56,57^. Moreover, an increased exponent is observed during response inhibition^58,59^. Increased cortical inhibition in adolescents observed here may, therefore, be associated with deficits in response inhibition caused by immature OFC-mediated outcome encoding.

In our analyses we focused on the theta frequency range because theta oscillations are hypothesized to modulate neural activity by coordinating activity across networks^60^ and may be important for organizing activity during active behavior^61^. Moreover, theta oscillations are hypothesized to reflect active learning^62^ and are associated with reward outcome expectancy^29^ and anticipation^63^. In our LFP data we found that adolescents exhibit stronger theta power in the DMS during premature actions. Stronger theta in adolescents following premature responding may therefore be associated with aberrant reward-expectation. Collectively, our electrophysiology data during CRIT demonstrate robust age differences in DMS activity after premature actions. These age-specific differences may reflect diminished capacity of the DMS to process action-outcome relationships in adolescents and premature actions in CRIT.

Because the adolescent frontal cortex and striatum are immature relative to adults, we tested whether that OFC–DMS synchrony would be reduced in adolescents. Unexpectedly, we observed stronger connectivity in adolescents and a significant positive relationship between neural synchrony and the number of premature actions. Recent findings in clinical populations suggest a positive relationship between impulsive behaviors and cortico-striatal connectivity strength. Patients with obsessive compulsive disorder, a condition characterized by increased compulsive actions, exhibit increased striatal-OFC connectivity and impulsivity when compared to healthy controls^64^. Furthermore, Sanefuji et al.^65^ classified children with ADHD as impulsive and inattentive subtypes and observed that impulsive but not inattentive children exhibit increased cortico-striatal connectivity. These data suggest that interactions between the OFC and DMS may play a causal role in age differences in impulsive actions. This notion is consistent with our chemogenetic experiments, where inhibition of OFC neurons that project to the DMS abolished age differences in premature actions. Collectively, these data suggest OFC–DMS connectivity plays a critical role in age associated differences in premature actions and that inefficient processing of task state in the OFC may influence the DMS’s ability to encode outcome-value information in adolescents. Given the increased premature actions of adolescents in CRIT, our OFC–DMS synchrony data may indicate that adolescents receive weaker feedback from their actions compared adults. In this way, their performance may be more habitual and less goal-directed, as others have hypothesized^66^.

### Limitations

Our current experimental design was guided by the hypothesis that adolescent and adult neurons are engaged differently during response inhibition. Given the short period of rodent adolescence, we were limited to employing a task that could be learned and completed in less than two weeks. We chose CRIT for this study, having extensively characterized it in adults and adolescents^21,67^. We, however, acknowledge that CRIT may not differentiate between learning and performance. Premature actions may result from an inability to learn the task contingency or a failure to inhibit a response, despite knowledge that inappropriate responding will not be rewarded. Thus, while our previous work had not shown any associative learning deficits in adolescents^68^, we cannot rule out that learning deficits in adolescents may have contributed to some of the observed behavioral age differences.

## Conclusions

We investigated the role of OFC–DMS in response inhibition in adolescent and adult rats. We observed a causal and age-dependent relationship in OFC projections to DMS, and pronounced age-dependent neuronal differences in the OFC and DMS. We hypothesize that these data reflect impaired state representation, likely due to reduced inhibition in adolescent OFC.

## Methods

*Subjects* subjects were male and female adolescent (PND 28–46; N = 19) and adult (PND 60+; N = 15) Long Evans rats bred in-house. Rats were pair housed until surgery under temperature and humidity-controlled conditions using a 12-h reverse light/dark cycle. Adolescents (PND 28) and adults (PND 60) were surgically implanted with custom-made 8-channel electrode arrays (50-µm-diameter tungsten wire insulated with polymide, California Fine Wire) in the lateral OFC (AP 3.2, ML 3.0, DV-4.0) and DMS (AP 0.7, ML 1.6,DV -4.0) under isoflurane anesthesia as described previously^19,20^. All animals had one week to recover before behavioral testing. All experiments were performed during the dark phase, in accordance with the National Institute of Health’s *Guide to the Care and Use of Laboratory Animals,* were approved by the Oregon Health and Science University Institutional Animal Care and Use Committee and were in compliance with the ARRIVE guidelines^69^. After the end of each experiment, animals were anesthetized, perfused with paraformaldehyde, and histology was performed to confirm probe placements (Supplementary Figure 3). Animals with probes outside of the target regions were excluded from analyses.

*Statistical analyses* all analyses were performed in MATLAB (MathWorks) and R (https://www.r-project.org/). Unless otherwise specified, all comparisons were first tested using analyses of variance (ANOVA) testing, followed by post hoc tests for multiple comparisons procedures.

*Behavior* all recordings took place in an operant chamber (Coulbourne, Instruments) equipped with a food trough and reward magazine opposite a nose-poke port with a cue light, infrared photo-detector unit, and a tone-generating speaker. Adolescents (PND 35–36) and adults (PND 67–68) were food restricted, habituated to the operant box and trained to nose poke in response to a light cue for a sucrose pellet (45 mg, Bio-Serv) on a fixed ratio one schedule over two days. After successful acquisition of cue-action responding, all animals begin CRIT training as described previously^21^. All CRIT sessions last 60 min. In each trial, in addition to the light cue that signaled that an action would lead to reward, animals were presented with an inhibitory cue (auditory tone). Responses made during the inhibitory cue were not rewarded and coded as “premature.” Following cessation of the inhibitory cue (variable time 5–30 s), the light cue remained lit for 10 s. An action executed during this time was rewarded and coded as “correct”. Failure to respond within 10 s was coded as an omission. Following either a response or omission, a variable length inter-trial interval of 10–12 s preceded the start of a new trial. All animals experienced ten CRIT sessions. Behavior data were analyzed using mixed design analyses of variance (ANOVA) testing with between subjects factor age and within subjects factor session.

*Electrophysiology recordings* single units and local field potentials (LFPS) were simultaneously recorded during performance of CRIT over the course of 10 sessions using a Plexon recording system. Spikes were amplified at 1000× gain, digitalized at 40 kHz, and single-unit data were band pass filtered at 300 Hz. Single units were isolated in Kilosort^70^. OFC neurons with baseline firing rates less than or equal to 10 Hz and spike widths greater than or equal to 0.30 were classified as putative pyramidal cells^71^. DMS neurons with baseline firing rates less than or equal to 5 Hz and spike widths greater than or equal to 0.25 were classified as putative medium spiny neurons^72^.

*Firing rate analyses* firing rates for all units were averaged across trials and Z-score normalized to each unit’s baseline (ITI prior to trial) firing rate. Friedman’s ANOVA was computed to determine whether firing rate changed during events of interest. Significant ANOVA results were followed up using Bonferroni corrected multiple comparisons. Age differences were assessed using permutation tests^73^.

*Spectral analyses* spectral analysis were performed on LFP recordings using the Matlab wrapper for the FOOOF toolkit^22^. A power spectrum density (psd) was computed using Welch’s power spectral density estimate and putative theta oscillation power was extracted. We did not compute power from datasets which did not exhibit putative theta oscillations, as indicated by a frequency specific peak. The aperiodic exponent or slope of the power spectral density was measured for all subjects. We used a broad frequency range (1–50 Hz) to assess the aperiodic fit and exponents as recommended^22,57^. The impact of age and event on the psd slope was first assessed using ANOVA testing. Significant main effects were followed up using Tukey HSD post doc testing. Significant interactions were followed up with Bonferroni corrected planned comparisons.

*Phase synchrony* data were filtered in the theta band (5–11 Hz) and segregated by behavioral epoch. To measure strength of OFC–DMS synchrony, the phase locking index γ was computed by taking the complex value of the average of all points (1/N) where ${\varphi }_{1}\left(t\right)$ and ${\varphi }_{2}\left(t\right)$ are two phases from the filtered signals, the phase difference $\theta \left({t}_{j}\right)={\varphi }_{1}\left({t}_{j}\right)-{\varphi }_{2}\left({t}_{j}\right)$, ${t}_{j}$ are the times of data points, and N is the number of all data points during the given time interval^31,74–76^. Age-group differences were assed using Welch’s two sample t-test. Person’s correlation was used to compare premature actions and synchrony across both groups.

*Chemogenetics* DREADD receptors were expressed in DMS projecting OFC neurons by injecting 0.5 µL of CAV/2 Cre (Montpellier Vector Platform) bilaterally in the DMS and 0.5 µL of the DREADD receptor AAV5-EF1a-DIO-hM4D(Gi)-mCherry (Addgene) in the OFC under isoflurane anesthesia on PND 20 as reported elsewhere^33^. Animals were divided into groups of either saline or CNO treatment and tested in either adolescence or adulthood. After two days of nose-poke training (PNDs 35–36, PND 58–59) animals experienced 10 days of CRIT with either CNO (10 mg/kg; Hello Bio) or saline treatments, 30 min prior to behavior. After the end of each experiment animals were anesthetized, perfused with paraformaldehyde, and the extent of virus expression was determined using immunohistochemistry techniques.

### Supplementary Information

Supplementary Figure 1.

Supplementary Figure 2.

Supplementary Figure 3.

## Data Availability

The datasets generated during and/or analyzed during the current study are available from the corresponding author on reasonable request. Data is provided within the manuscript or supplementary information files. Codes will be made available upon request.
