# Supplementary figures and images for: Adolescent rats engage the orbitofrontal-striatal pathway differently than adults during impulsive actions
Source: Sci Rep. 2024 Apr 13;14:8605. doi: 10.1038/s41598-024-58648-w (PMC11016110; doi:10.1038/s41598-024-58648-w)

A

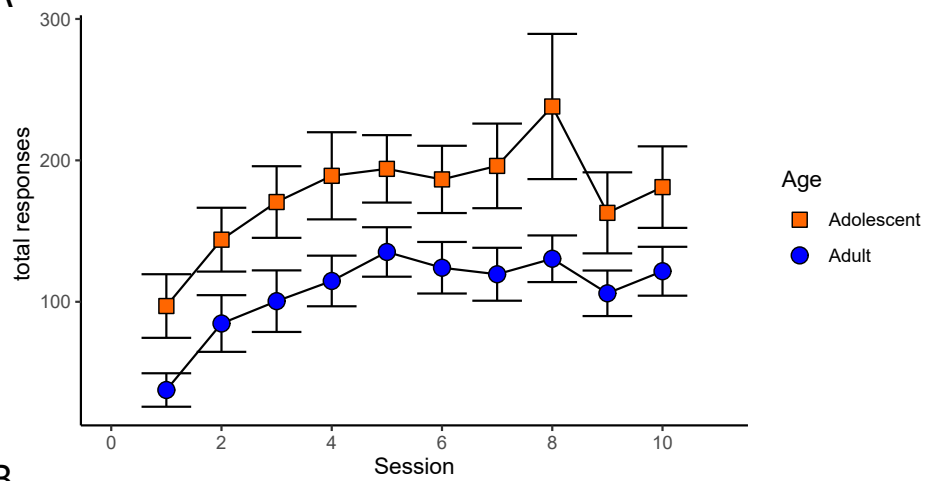

B

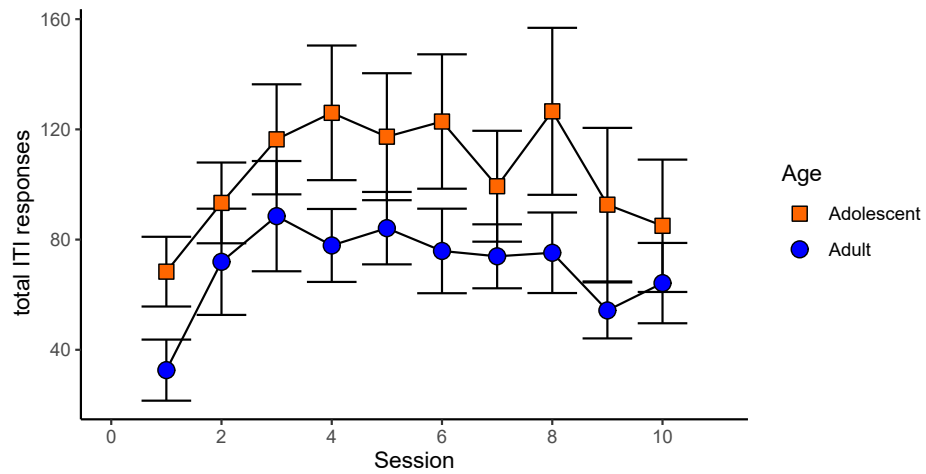

C

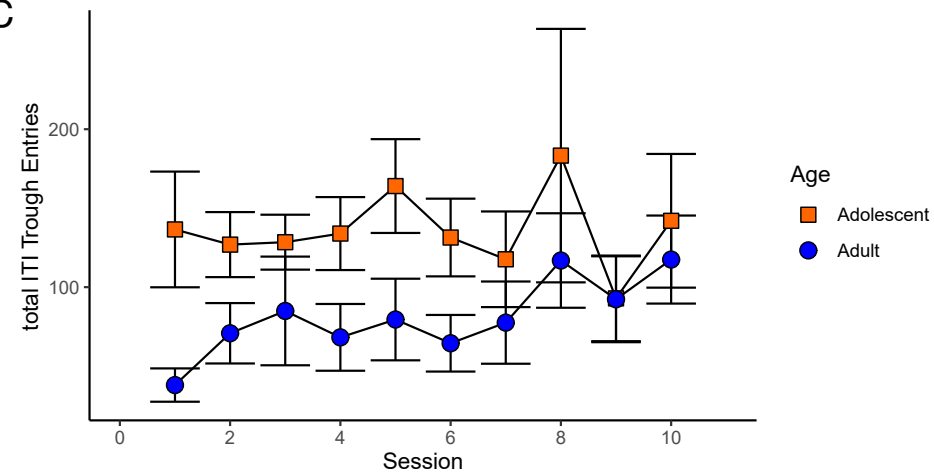

Supplement: Supplementary file 2 — Supplementary Figure 2. [file 41598_2024_58648_MOESM2_ESM.pdf]

+4.20

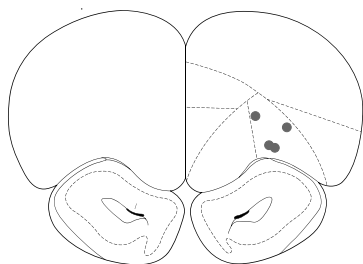

+1.70

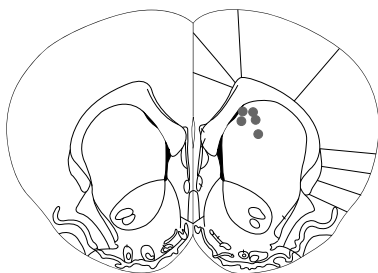

+3.70

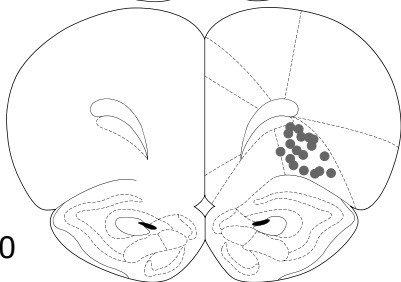

+1.60

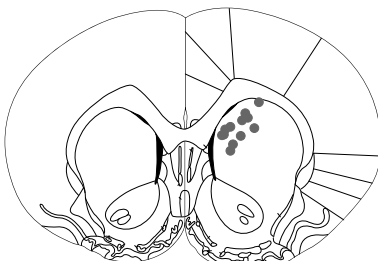

+3.20

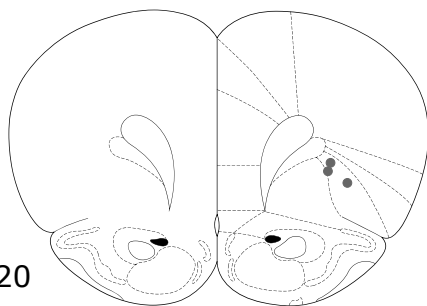

+1.20

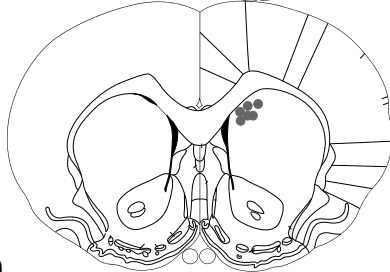

Supplement: Supplementary file 3 — Supplementary Figure 3. [file 41598_2024_58648_MOESM3_ESM.pdf]
