# Supplementary material for: Adolescent rats engage the orbitofrontal-striatal pathway differently than adults during impulsive actions
Source: Sci Rep. 2024 Apr 13;14:8605. doi: 10.1038/s41598-024-58648-w (PMC11016110; doi:10.1038/s41598-024-58648-w)
Supplement: Supplementary file 1 — Supplementary Figure 1. [file 41598_2024_58648_MOESM1_ESM.pdf]

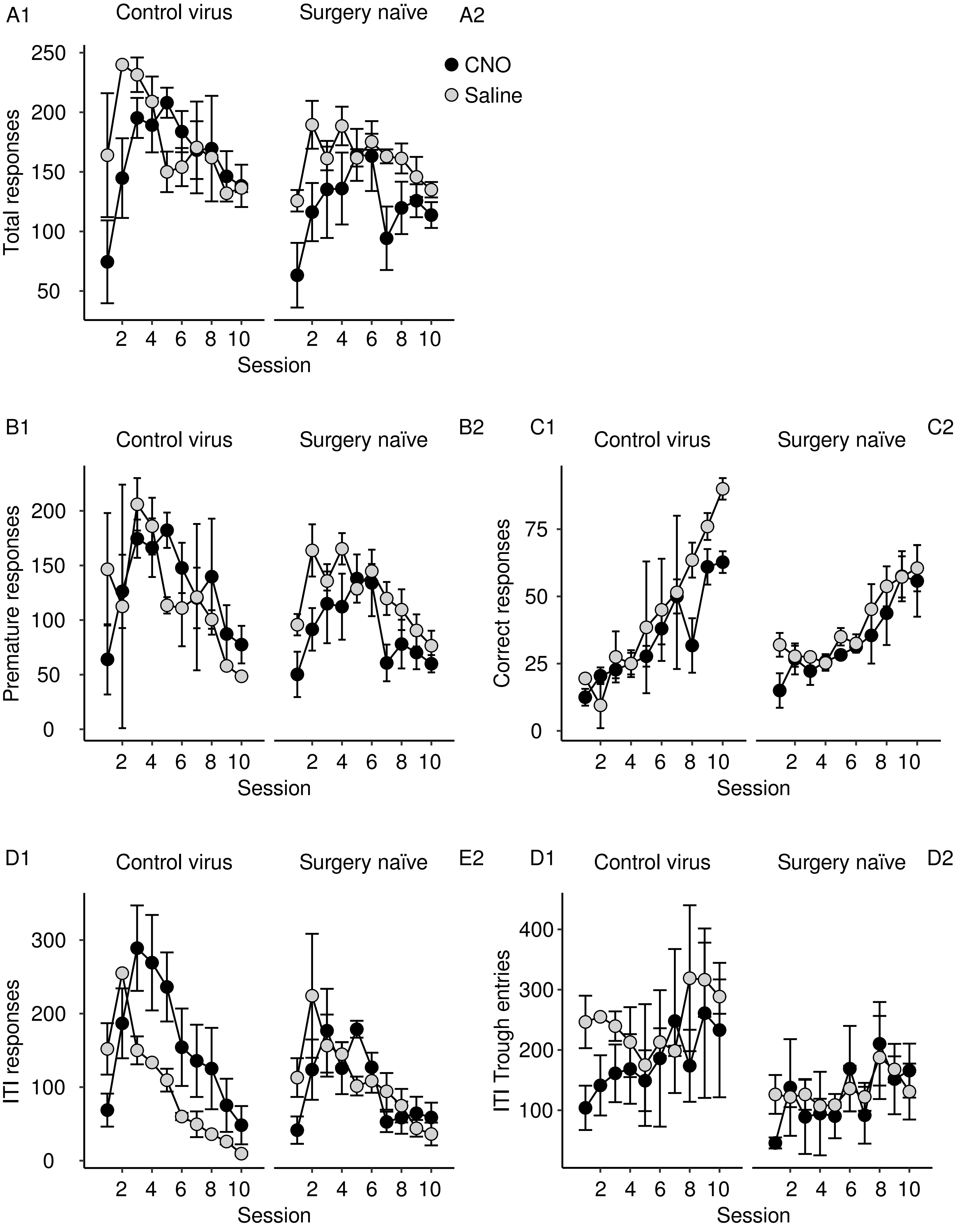
A1
Control virus
Surgery naïve
A2
CNO
Saline
Total responses
250
200
150
100
50
2
4
6
8
10
Session
B1
Control virus
Surgery naïve
B2
Premature responses
200
150
100
50
0
Session
C1
Control virus
Surgery naïve
C2
Correct responses
75
50
25
0
Session
D1
Control virus
Surgery naïve
E2
ITI responses
300
200
100
0
Session
D1
Control virus
Surgery naïve
D2
ITI Trough entries
400
300
200
100
Session
